# A Practical Treatise on Dental Medicine: Being a Compendium of Medical Science as Connected with the Study of Dental Surgery

**Published:** 1851-04

**Authors:** 


*A Practical Treatise on Dental Medicine: being a Compendium of Medical Science as connected with the Study of Dental Surgery.* By Thos. E. Bond, A. M., M. D., Professor of Special Pathology and Therapeutics in the Baltimore College of Dental Surgery. 8vo., pp. 324. Philadelphia: Lindsay & Blakiston, 1851.

Until within the last few years, dental surgery was almost universally regarded as a mere mechanical art—as having no connection whatever with general medicine, and as requiring only a very small amount of manual dexterity for its successful practice. Hence the duties of the profession, as might have been supposed, were, for the most part, exercised in the rudest and most bungling manner. With few exceptions, the teeth were treated as isolated, inorganic bodies—as having neither anatomical, physiological nor pathological relationships with other parts of the body. But, under the influence of more liberal and enlightened views, dentistry has rapidly advanced from this humble and lamentable position, until it is now regarded as an important branch of the curative art; and that for the successful practice of which, as thorough a training is required as for the practice of any other specialty of medicine. The connecting link between it and general medicine, however, has, up to the present time, been wanting. But this is now supplied, and if not as fully as could have been desired, yet in a manner that must be highly gratifying both to the members of the medical and dental professions.

The work of Professor Bond opens a new era in the history of dental surgery, pointing out, as it does, the pathological relationship between diseased teeth and other parts of the body, it demonstrates, in the fullest and most conclusive manner, the importance and absolute necessity of a thorough knowledge of *anatomy, physiology, pathology* and *therapeutics*, to the dentist.

Indeed, we regard the book as indispensable, both to the student and practitioner of dental surgery; and it may be read with equal profit by the physician. It embodies, and within narrow limits, a large amount of valuable information, embracing etiology; symptoms of disease; diagnosis; treatment of disease; nature of disease; ulcers, depending both upon local and constitutional causes; tumors; diseases of the teeth and face, dependent upon morbid conditions, either general or of other parts; morbid secretions of the mouth; morbid effects of conditions of the teeth, and the parts immediately connected with them, upon the general system; morbid effects of first dentition; sympathetic diseases of dentition; effects of diseased teeth and gums upon the general health; wounds of the mouth and face; particular affections of the mouth and adjacent parts; diseases of the lips; diseases of the glands and gland ducts; tumors requiring amputation of a part or of the whole of the upper jaw; amputation of the lower jaw; diseases of the antrum, and diseases of the palate.

As a literary production, the work is highly creditable to the accomplished author; and as a contribution to the science of dental surgery, it is certainly invaluable.

